# Bacterial glucuronidase as general marker for oncolytic virotherapy or other biological therapies

**DOI:** 10.1186/1479-5876-9-172

**Published:** 2011-10-11

**Authors:** Michael Hess, Jochen Stritzker, Barbara Härtl, Julia B Sturm, Ivaylo Gentschev, Aladar A Szalay

**Affiliations:** 1 Department of Biochemistry, Biocenter, University of Würzburg, Würzburg, Germany; 2 Genelux GmbH, Bernried, Germany; 3 Genelux Corporation, San Diego, CA, USA; 4 Department of Radiation Oncology, Moores Cancer Center, University of California, San Diego, La Jolla, CA, USA

**Keywords:** beta-glucuronidase, oncolytic virus, cancer, reporter, fluorescent probe

## Abstract

**Background:**

Oncolytic viral tumor therapy is an emerging field in the fight against cancer with rising numbers of clinical trials and the first clinically approved product (Adenovirus for the treatment of Head and Neck Cancer in China) in this field. Yet, until recently no general (bio)marker or reporter gene was described that could be used to evaluate successful tumor colonization and/or transgene expression in other biological therapies.

**Methods:**

Here, a bacterial glucuronidase (GusA) encoded by biological therapeutics (e.g. oncolytic viruses) was used as reporter system.

**Results:**

Using fluorogenic probes that were specifically activated by glucuronidase we could show 1) preferential activation in tumors, 2) renal excretion of the activated fluorescent compounds and 3) reproducible detection of GusA in the serum of oncolytic vaccinia virus treated, tumor bearing mice in several tumor models. Time course studies revealed that reliable differentiation between tumor bearing and healthy mice can be done as early as 9 days post injection of the virus. Regarding the sensitivity of the newly developed assay system, we could show that a single infected tumor cell could be reliably detected in this assay.

**Conclusion:**

GusA therefore has the potential to be used as a general marker in the preclinical and clinical evaluation of (novel) biological therapies as well as being useful for the detection of rare cells such as circulating tumor cells.

## Background

The regained interest in oncolytic viruses over the past several years led to an enormous leap in the field with more and more oncolytic viruses to be described and yet to come. Not only were those viruses genetically altered to attenuate their virulence, to improve their safety profile and enhance their tumor specificity, but they also were equipped with additional genes for e.g. cytotoxins, cytokines, prodrug converting enzymes and reporter genes that improved the overall performance of these viruses [1,2]. Among those, vaccinia virus is one of the most promising candidates and has several advantages: Since this large DNA virus encodes e.g. its own DNA polymerase it is able to replicate in the cytoplasm of infected host cells thereby minimizing the risk of DNA integration into the host genome. Moreover, vaccinia virus displays a broad host cell range, rapid spread and a high capacity (up to 25 kbp) for genetic payload of foreign DNA [3]. Of note and importance regarding the safety of vaccinia virus, is also its billion-fold use in humans during the eradication program of smallpox, as well as the fact that vaccinia virus is not a human pathogen. On top of that, recombinant vaccinia virus strains (rVACVs) specifically colonize solid tumors in mice while not infecting other organs [4-7]. Therefore, its use in human patients was pursued and first human trials have already been carried out successfully [8-11].

A reliable monitoring of successful tumor colonization in humans would have an enormous impact not only on clinical trials, but also to predict possible outcomes of oncolytic virus therapy. In this aspect, we and others used different kind of reporter genes for optical (e.g. [5]), or radiological (e.g. [12-15]) imaging modalities. This enabled visualization of virus replication within live animal models. However, as optical imaging has strong limitations in penetration depth and radiological imaging is time consuming and requires the need of specialized personnel and expensive equipment, a cheap and simple method with short turn-around-times is urgently needed. In particular, if this method could also be used in other biological therapy approaches.

Beta-glucuronidases catalyze the hydrolysis of ß-D-glucuronides into the corresponding D-glucuronate and alcohol. While the mammalian enzymes with a pH-optimum under acidic conditions (pH 4 to 5) have strongly reduced capacity at normal (neutral) tissue pH, the *E. coli *enzyme encoded by *gusA *works optimal in the range of pH 6.8 to 7.7 [16].

Its first use as a (fusion-)reporter gene was described by Jefferson et al. [17,18] and was extensively used in plant physiology studies. In mammals, bacterial glucuronidase was mainly used in prodrug studies, due to the very low abundance of human glucuronidase in human serum [19]. Several strategies were successfully employed: e.g. fusion of cancer specific antibody-fragments with beta-glucuronidase [20] or tumor selective expression of the enzyme using bacteria [21] or adenoviruses [22,23]. The reporter gene properties of the enzyme were not studied as extensively in animals. However, two independent approaches were published that looked at the potential of using beta-glucuronidase as a target structure for radiotracers in positron-emission-tomography [24,25]. In another study, a membrane-anchored form of a mouse-glucuronidase was used in combination with the fluorescein di-beta-D-glucuronide (FDGlcU) which was hydrolyzed to a fluorescent reporter that could be used to assess the location and persistence of gene expression in vivo [26].

Here, we show that beta-glucuronidase in combination with fluorogenic substrates cannot only be used for localization of enzyme expression, but also as a general biomarker for foreign protein expression in serum samples. Consequently, the described test-system could be applied to all kinds of biological therapies which depend on heterologous gene expression.

## Materials and methods

For evaluation of the described glucuronidase assay it was necessary to confirm the heterologous gene expression of the described vaccinia virus strain by Western blot analysis as well as immuno-staining studies in cell culture and infected tumor sections. The assay itself (described in the "Fluorogenic probes and detection of fluorescence products" section) was tested with purified enzyme as well as with samples from vaccinia virus injected animals.

### Cell culture

Human A549 lung cancer cells (ATCC No. CCL-185) were cultured in RPMI-1640 medium containing 10% fetal bovine serum (FBS) and 1% antibiotic-antimycotic solution (PAA Laboratories, Cölbe, Germany) under standard cell culture conditions (37°C, 5% CO_2_). MTH52c is derived from a malignant small-cell canine carcinoma of the mammary gland [27] and cultured in DMEM supplemented with antibiotic-antimycotic solution and 20% FBS.

### Vaccinia viruses

The attenuated vaccinia virus strain GLV-1h68 was purified as previously described [4]. For generation of control viruses, *lacZ *and *gusA *of GLV-1h68 were replaced by nonrelevant gene constructs to create viruses negative for beta-galactosidase (rVACV-LacZ^-^) and beta-glucuronidase (rVACV-GusA^-^) respectively (Additional File 1, Figure S1).

### Infection of cell cultures

Two days before infection, cells were seeded in 6-well plates for western blot analysis or 12-well plates containing sterile cover slips for microscopy studies. 90% confluent cell layers were either mock treated or infected with GLV-1h68, rVACV-LacZ^- ^or rVACV-GusA^- ^at a multiplicity of infection (MOI) of 0.1 for 1 h at 37°C and 5% CO_2 _in medium containing 2% FBS. Afterwards the infection medium was aspirated and replaced by standard cell culture medium.

### Western blot analysis

For detection of proteins, infected cells were harvested and lysed in SDS sample buffer at 6, 12, 24 and 48 hours post-infection (hpi). Lysates were separated by 10% SDS-Polyacrylamide gel electrophoresis and subsequently transferred onto a nitrocellulose membrane (Whatman GmbH, Dassel, Germany). After blocking in 5% skim milk in PBS, the membrane was incubated with anti-beta-glucuronidase rabbit polyclonal antibody (G5420, Sigma-Aldrich, Schnelldorf, Germany), anti-beta-galactosidase rabbit polyclonal antibody (A-11132, Molecular Probes, Leiden, Netherlands), anti-GFP rabbit polyclonal antibody (sc-8334, Santa Cruz, Heidelberg, Germany) or anti-beta-actin mouse monoclonal antibody (ab6276, Abcam, Cambridge, UK). The first antibodies were detected using horseradish peroxidase-conjugated anti-mouse (ab6728, Abcam, Cambridge, UK) or anti-rabbit (ab6721, Abcam, Cambridge, UK) secondary antibodies, followed by enhanced chemiluminescence detection.

### X-Gal/X-GlcU staining and microscopy studies

For the analysis of expression and activity of beta-galactosidase and beta-glucuronidase respectively, A549 cells were seeded on coverslips and infected with 200 PFU GLV-1h68, rVACV-LacZ^- ^or rVACV-GusA^- ^per well. After incubation for 2 days, cells were fixed using 4% paraformaldehyde and washed twice in PBS. Staining solutions consisted of 40 μl X-Gal (5-bromo-4-chloro-3-indolyl-β-D-galactoside, Invitrogen, Karlsruhe, Germany) and X-GlcU (5-bromo-4-chloro-3-indolyl-β-D-glucuronide, Invitrogen, Karlsruhe, Germany) respectively in dimethylformamide (40 mg ml^-1^), ferricyanide (12 mM K_3_Fe(CN)_6_), 5.2 mM MgCl_2 _and ferrocyanide solution (12 mM K_4_Fe(CN)_6_). Coverslips were stained with either X-Gal or X-GlcU solution and incubated for 24 h at 37°C before mounting in Mowiol. Images were taken with a Zeiss Axiovert 200 M microscope.

### Histology and immunofluorescence

For histological analysis, snap-frozen tumors were fixed in 4% paraformaldehyde/PBS overnight at 4°C. Samples were embedded in 5% (w/v) low-melt agarose (AppliChem, Darmstadt, Germany) und 100 μm sections were cut using a Leica VT1000S Vibratome (Leica, Heerbrugg, Switzerland) as described before [28]. After permeabilizing in 0.3% Triton X-100/PBS, sections were incubated with Hoechst 33342, anti-beta-glucuronidase rabbit polyclonal antibody (G5420, Sigma-Aldrich, Schnelldorf, Germany) and anti-beta-galactosidase chicken polyclonal antibody (ab9361, Abcam, Cambridge, UK) before staining with Cy-5-conjugated donky anti-rabbit and Cy-3 conjugated donky anti-chicken secondary antibodies (Jackson ImmunoResearch, West Grove, PA). Mowiol-embedded sections were examined using a Leica MZ 16 FA Stereo-Fluorescence Microscope equipped with a Leica DC500 Digital Camera. Digital Images were processed with Photoshop 7.0 (Adobe Systems, San Jose, CA) and merged to yield pseudocolored pictures.

### Animal studies

A549 and MTH52c xenograft tumors were developed in 6- to 8-week-old nude mice (NCI:Hsd:Athymic Nude *Foxn1*^nu^, Harlan Borchem, Germany) by implanting 5 × 10^6 ^cells subcutaneously in the right abdominal flank. Two to three weeks after implantation, mice were anesthetized with isoflurane and injected with a viral dose of 5 × 10^6 ^PFU GLV-1h68, rVACV-LacZ^- ^or rVACV-GusA^- ^in 100 μl PBS via the retro-orbital sinus vein.

Blood and urine collection of mice was carried out under anesthesia by a heparinised capillary pipet (No. 554/20, Assistent, Sondheim, Germany) via the retro-orbital sinus vein for blood and a bladder catheter (No. 381312, Becton Dickinson, Heidelberg, Germany) for urine respectively.

All animal experiments were carried out in accordance with protocols approved by the Regierung von Unterfranken (Würzburg, Germany) (protocol number AZ 55.2-2531.01-17/08) and/or the the Institutional Animal Care and Use Committee (IACUC) of Explora BIOLABS, located in San Diego Science Center (San Diego, USA) (protocol number: EB08-003).

### Fluorogenic probes and detection of fluorescence products

The lyophilized fluorogenic probes FDGlcU, FDG and 4-Methylumbelliferyl-b-D-glucuronide (4-MUG) (Invitrogen, Karlsruhe, Germany) were dissolved in DMSO (36.5 mM). For in-vivo studies, 5 μl of each stock dilution was mixed with 195 μl PBS and injected intraperitoneally. Whole body and urine fluorescence analysis was performed using a Maestro EX imaging system (CRI, Woburn, MA). For serum analysis, the collected mouse serum was diluted 1:15 with PBS and 80 μl of each sample were mixed with of either 2.5 μg FDGlcU or 1.5 μg 4-MUG if not otherwise indicated. Human serum of healthy individuals (Zen-Bio Inc, Research Triangle, NC) was obtained from whole blood and 10 μl were used in the described assay. After incubation for 1 h at 37°C (if not otherwise indicated), fluorescence was read in Lumox 384-well plates (Sarstedt, Nümbrecht, Germany) using an Infinite 200 Pro Microplate Reader (Tecan, Crailsheim, Germany) or a Spectra Max M5 (Molecular Devices, Sunnyvale, USA) and fluorescence intensities are listed as relative fluorescence units.

## Results

Biological therapies, like e.g. oncolytic virotherapy, are dependend on the expression of genes that are not functionally expressed in the respective patient. We recently described the use the rVACV strain GLV-1h68 in combination with a prodrug seco-analog of duocarmycin SA which is activated by the virus encoded beta-galactosidase [29]. Although very promising results were observed in cell culture, the synergistic effect in tumor bearing mice were less pronounced, which was also true for a bacterial glucuronidase activatable version of the prodrug (Hess et al, unpublished). Among other potential reasons for this observation was a non-favorable pharmacokinetic of the prodrugs. Therefore, we wanted to analyze the activation and pharmacokinetics of other beta-galactosidase or glucuronidase substrates that could actually be visualized by optical imaging.

**Figure 1:**
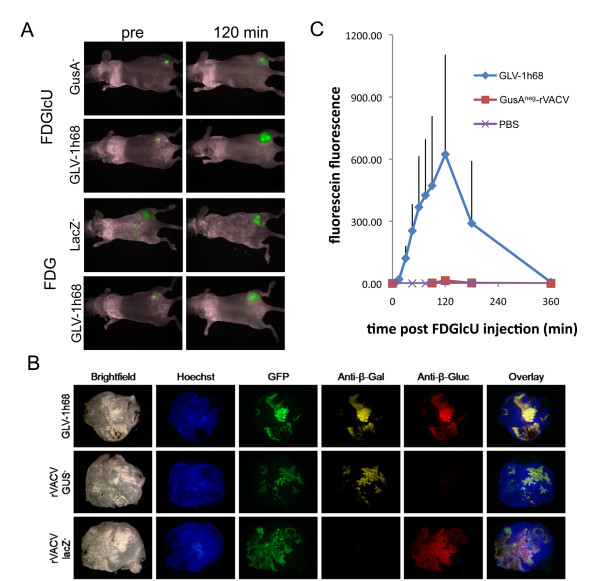
**Fluorogenic compound activation in rVACV-colonized tumors**. A) Two hours post intraperitoneal injection of FDG and FDGlcU tumor specific fluorescein dependent fluorescence was observed in the presence of LacZ- and GusA-expressing vaccinia virus GLV-1h68 respectively. In rVACV-LacZ-negative and rVACV-GusA-negative colonized tumors, only GFP fluorescence was observed (no difference in fluorescence between pre- and 120 min post fluorogenic compound injection). B) Fluorescence immunohistochemical analysis of GLV-1h68, rVACV-GusA-negative and rVACV-LacZ-negative colonized tumors. Blue - Hoechst 33342 stained DNA, green - virus encoded GFP, yellow - beta-galactosidase, red - beta-glucuronidase. C) Tumor specific fluorescence over time post FDGlcU-injection (n = 4; average plus standard deviation is shown).

As fluorescent probes can be directly detected in small animals, the compounds FDG (substrate for the beta-galactosidase LacZ) as well as FDGlcU (a glucuronidase substrate) were injected in tumor bearing mice that had previously been injected with oncolytic rVACV (GLV-1h68) encoding both enzymes. Upon cleavage the probes were converted to fluorescein which resulted in a change of their fluorescent properties that was readily detectable with a small animal fluorescence imaging system (Figure 1A). Animals that were previously injected with PBS or the control rVACV strains not expressing beta-galatosicase (rVACV-LacZ^-^) and glucuronidase (rVACV-GusA^-^) respectively served as controls. It became evident that each fluorogenic probe could indeed be activated in the tumor and that this activation is dependent on the expression of LacZ and GusA respectively (Figure 1A and 1C). The maximum fluorescein fluorescence in the tumor was observed 120 min after intraperitoneal injection (Figure 1B). Upon intravenous FDGlcU-injection the maximum fluorescence was already observed 20 min post injection (Additional File 2, Fig. S2). About 6 hours post injection (hpi), the GFP-dependent fluorescence remained while most of the compound specific fluorescence was gone (Figure 1A and 1B).

Consequently it was investigated how the produced fluorescein was removed from the tumor. Possible explanations could be fluorescein instability in or compound excretion from the tumor.

In favour of (renal) excretion was the presence of fluorescein in the urine of injected mice. Moreover, when fluorescein itself was injected directly into the tumor the compound accumulated in the bladder and was then secreted with the urine (data not shown) while it disappeared from the tumor.

**Figure 2:**
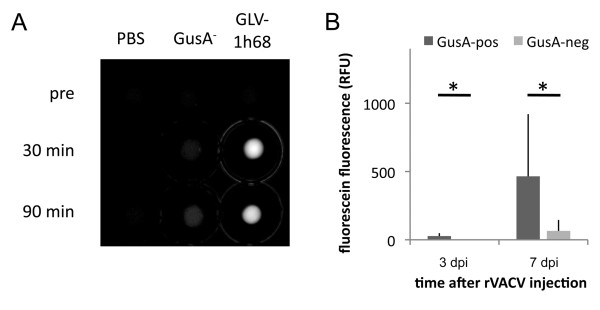
**Analysis of urine samples derived from mice before, 30 min and 90 min post FDGlcU injection respectively**. A) Five μl urine were analyzed in the Maestro Imaging system. Unmixed fluorescein specific fluorescence is shown. B) Three and seven days post rVACV injection urine was sampled before and 90 min post FDGlcU injection. Average plus standard deviation of fluorescein specific fluorescence of GusA-positive (n = 6 for 3 dpi and n = 8 for 7 dpi) and GusA-negative (n = 4) rVACV colonized tumors. * indicates p < 0.05.

The presence of the activated probe in the urine of GLV-1h68 injected tumor bearing mice offered the possibility to evaluate this as a possible biomarker for successful tumor colonization. To test this, mice were anesthesized and urine was isolated via a bladder cathether before and 90 minutes after i.p. injection of FDGlcU. Indeed, fluorescein was present in urine of GLV-1h68 treated tumor bearing animals but could not be observed in FDGlcU injected control mice that either had non-colonized or rVACV-GusA^- ^colonized tumors (Figure 2A, B). Therefore, one could establish a test for the presence of GLV-1h68 in tumors of live mice with a simple urine test after systemic injection of FDGlcU. Nevertheless, we did not proceed to establish a urine based test system as the injection of FDGlcU in human patients seemed unlikely.

**Figure 3:**
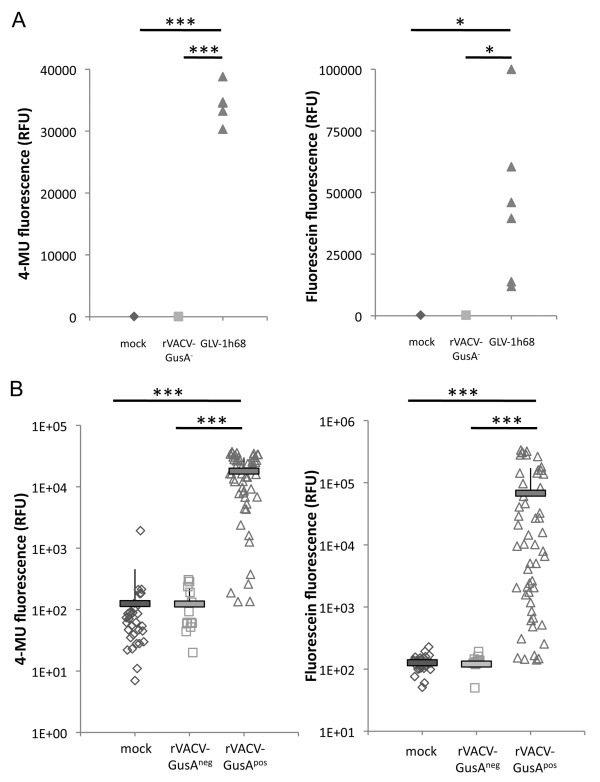
**Glucuronidase specific fluorigenic compound activation in serum of tumor bearing mice**. A) Tumor bearing mice were mock injected (n = 2) or injected with GLV-1h68 (n = 6) and rVACV-GusA-negative (n = 6) respectively. Seven days later 5 μl serum was co-incubated for 1 h at 37°C with 4-MUG and FDGlcU respectively and subsequently specific fluorescence was determined. B) Retrospective serum analysis. Serum samples (n = 99) from different tumor xenograft models (GI-101A, A549, DU-145, PANC-1, HT-29) were retrospectively tested. Samples were derived from mock (n = 33) injected mice or mice treated for different periods of time (7 to 53 days) with several GusA-positive (n = 53) or -negative (n = 13) rVACV. * indicates p < 0.03; *** indicates p < 0.001.

Although fluorescein obviously was renally excreted, it was also investigated whether active enzymes that were specifically produced in the tumor tissue leaked out and were then present in the serum of GLV-1h68 injected tumor bearing mice leading to additional (non-tumor specific) cleavage of FDGlcU. To investigate this, the serum of tumor bearing mice which were previously injected with the GusA encoding strain GLV-1h68 was incubated with FDGlcU or 4-MUG. Both substances can be hydrolysed by glucuronidase to the fluorescent products fluorescein and 4-MU respectively. As latter has excitation and emission maxima of 365 nm and 455 nm respectively, it was not suitable for optical *in vivo *imaging and therefore only used in *in vitro *studies. In our experiments, no fluorescence was observed when mice were injected with PBS or GusA-negative control rVACV while the fluorescent compounds clearly were detectable after co-incubation with serum derived from GLV-1h68 injected mice (Figure 3A). The serum therefore contained active (non-secreted, but shed) enzymes that were produced in the tumor tissue.

Theoretically, this does pave the way to a simple blood test that could be used to distinguish tumor bearing from healthy patients and/or to distinguish between successful from unsuccessful tumor colonization of GusA-encoding oncolytic virus strains in cancer bearing patients. To confirm this, we retrospectively tested serum samples (n = 99) from different tumor xenograft models (GI-101A, A549, DU-145, PANC-1, HT-29) that were collected in our lab over about 4 years from mice injected with PBS (n = 33) or treated for different periods of time (7 to 53 days) with several GusA-positive (n = 53) or -negative (n = 13) rVACV (Figure 3B). The test of these probes confirmed a significant (p < 0.001) difference between the GusA containing group and those that did not result in GusA-production. As the test was done in retrospect, we could not confirm/exclude successful tumor colonization in those mice which had GusA-negative results in the blood test. This could explain the negative results for 5 of the GusA-rVACV injected tumor bearing mice.

**Figure 4:**
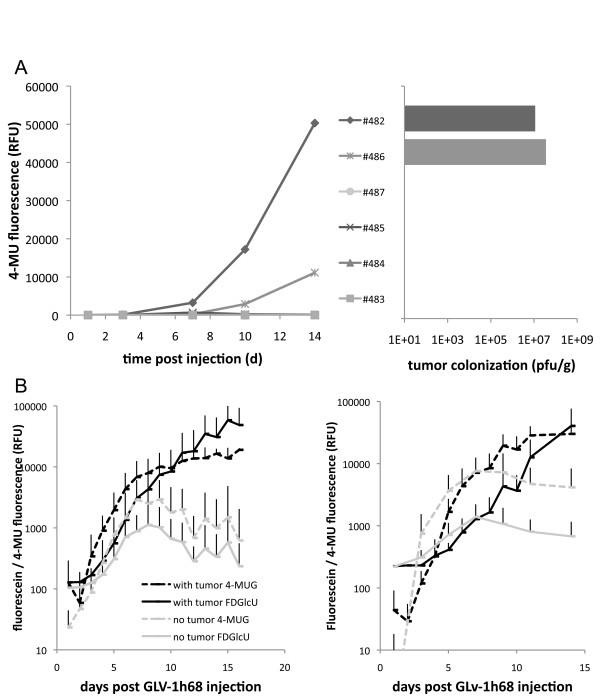
**Signal generation is both dependent on presence of tumors and *gusA*-encoding rVACV**. A) Confirmation of successful tumor colonization after low-dose injection of 1 × 10^5 ^pfu GLV-1h68. In the left panel time dependent 4-MU fluorescence is shown for individual sera of rVACV injected tumor bearing mice. The sera of two mice (#482 and #486) resulted in generation of significant amounts of 4-MU. Viral titer analysis of tumors from the same mice revealed that only those mice (#482 and #486) had virus colonized tumors which also resulted in generation of 4-MU. B) Tumor bearing (black) and non-tumor bearing (grey) control male (left panel) and female (right panel) mice were injected with 5 × 10^6 ^pfu GLV-1h68 into the retro-orbital sinus vein. Analysis of sera revealed conversion of the fluorigenic compounds FDGlcU (solid lines) and 4-MUG (broken lines) in all mice. Significant differences (p < 0.05) between tumor bearing and control mice was observed after 9 dpi. Results show average plus standard deviation of fluorescein and 4-MU fluorescence.

Next, it was investigated whether one could confidently decide via the blood test if a tumor was colonized or not. Tumor bearing mice (n = 5) were systemically injected with a low dose (1 × 10^5 ^PFU) of GLV-1h68. This dose was known to result in colonization of only some tumors while other tumors do not get colonized at all. Serum was then isolated 1, 3, 7, 10 and 14 days later, before mice were sacrificed and tumor colonization was tested by conventional plaque assay confirming the results observed by the FDGlcU/4-MUG based blood test (Figure 4A). The very same 2 out of 5 mice were found to have GLV-1h68 in their tumors as well as being positive in the FDGlcU/4-MUG based blood tests.

In another experiment we tested the suitability of the blood test to differentiate between tumor bearing and control tumor free mice. Unexpectedly, seven days post injection of GLV-1h68 in non-tumor bearing mice low but significant glucuronidase activity was detected in the serum of tumor free mice. Closer examination of those mice revealed GFP expression in the paws of 2 mice (data not shown). Therefore, the mice were sacrificed and a plaque assay of several organs was used to find the origin of glucuronidase production (Table 1). Apart from the two infected paws virus was reproducibly isolated in low concentration from ovaries of non-tumor bearing mice. Interestingly, ovaries of tumor bearing mice were essentially free of virus.

**Table 1:** Viral distribution in tumor bearing and non-tumor bearing mice 14 dpi

pfu/g tissue										fluorescence	
mouse #	tumor	blood	ovaries	spleen	kidneys	liver	lung	brain	paw	4-MU	fluorescein
1	NA	ND	ND	ND	ND	ND	ND	ND	2.10E+06	8599	1179
2	NA	ND	2500	ND	ND	ND	ND	100	ND	4418	607
3	NA	ND	5300	ND	ND	ND	ND	20	ND	6775	855
4	NA	ND	12000	ND	100	ND	ND	ND	5.00E+05	14587	2641
5	NA	ND	8800	ND	ND	100	ND	ND	ND	4325	483
6	NA	ND	ND	ND	ND	0	ND	ND	ND	5272	667
7	2.70E+07	ND	ND	40	ND	ND	20	ND	ND	40812	168977
8	5.15E+07	ND	ND	100	ND	ND	ND	ND	ND	43732	61137
9	2.65E+07	ND	ND	ND	100	ND	ND	ND	ND	43866	43572
10	9.00E+06	ND	20	20	100	ND	80	ND	ND	38449	135454
11	8.50E+06	ND	ND	ND	20	ND	ND	ND	ND	41645	28754

NA - not applicable. ND - not detectable. Detection limit 20 pfu/g.

Background expression of glucuronidase in healthy subjects would of course exclude using this test system for detection of tumors if no difference existed that allowed differentiation between cancer patients and healthy individuals. Time course studies in male (n = 12 tumor bearing and 12 tumor free) and female mice (n = 24 tumor bearing and 6 tumor free) over a period of 14-16 days in which blood was taken every other day (in one half of the mice blood was taken on even, in the other on uneven days post virus injection), again showed low levels of glucuronidase present in the serum of tumor free mice. These were similar to those observed in tumor bearing mice until 8 days post virus injection (Figure 4B and Additional File 3, Fig. S3). After that however, significant changes occurred. While the glucuronidase activity in the serum of tumor free mice decreased, the fluorescence signal in the tumor bearing animal probes increased.

Taken together, 9 days after injection of the virus it was possible to decide with confidence whether A) an existing tumor was successfully colonized and/or B) a tumor was present in the *gusA *encoding rVACV injected mouse.

In a next step the sensitivity and performance of the described test was evaluated in *in vitro *studies regarding its clinical translatability. We found a positive correlation between the fluorescence signal intensities and increasing A) glucuronidase concentration, B) substrate (FDGlcU or 4-MUG) concentration and C) incubation time (Additional File 4, Fig. S4). The data also revealed that very low glucuronidase concentrations could be detected using the fluorogenic FDGlcU or 4-MUG substrates which should allow detection of lysed tumor cells not only in mice but also in humans. Feasibility in the presence of human serum was shown by running the assay in the presence or absence of 10 μl human serum (Additional File 5, Fig. S5). The data revealed that neither the sensitivity nor the fluorescence intensity of the assay was changed in the presence of human serum.

**Figure 5:**
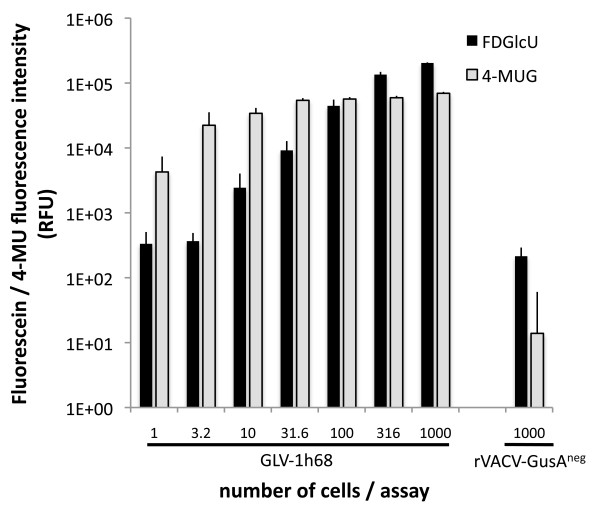
**Minimal amount of GLV-1h68 infected cancer cells necessary for positive detection**. A549 cells were infected with GLV-1h68 or control-rVACV (rVACV-GusA^neg^). One day later, the amount of infected cells was determined by flow cytometry and the cells were seeded in half-log dilutions in 384-well plates with concentrations varying from 1.0 to 1000 cells/well and co-incubated with FDGlcU and 4-MUG respectively. Data represent average plus standard deviation (n = 6).

As the presence of glucuronidase relies on the production by infected cancer cells, the minimal amount of infected cancer cells was tested that was needed to generate a positive fluorescent signal. For this, A549 cells were infected with GLV-1h68 or control-rVACV. One day later, the amount of infected cells was determined by counting and flow cytometry and the cells were diluted and seeded in half-log dilutions in 384-well plates with concentrations varying from approx. 1.0 to 1000 infected cells/well and co-incubated with 6.3 μg FDGlcU and 3.4 μg 4-MUG respectively. To obtain high sensitivity, the probes were incubated at 37°C over night and analyzed the next day (Figure 5). Surprisingly, even a single infected cancer cell could be readily detected using this test when using 4-MUG as a substrate. This allowed us to calculate the potential sensitivity of the described test for the detection of tumors in human patients using two different approaches: 1) Assuming that more serum (e.g. 50 μl) would be used when testing the system on human patient samples, and an average total blood volume of about 4.7 liters, only approx. 10^5 ^infected cancer cells would be sufficient to generate a detectable fluorescent signal. 2) The fluorescent signal generated from a single cancer cell was similar to that obtained from 0.2 units glucuronidase. Increasing the sensitivity by adding more fluorescent substrate 0.05 units glucuronidase were easily detectable, corresponding to a concentration of 1 unit glucuronidase/ml serum (again using 50 μl serum/test) or 4700 units/average blood volume of a human patient. Therefore, as low as 2.4 × 10^4 ^infected cancer cells would be sufficient for a positive signal.

## Discussion

Biological therapies including stem cell therapy, gene therapy, immunotherapy, oncolytic virotherapy etc. are gaining more and more impact in e.g. regenerative medicine, immunology, oncology and treating various diseases. All of these require the expression of certain genes that usually are not or only weakly expressed in the targeted tissue. While progress in terms of biological effectiveness is made in each field, no common reporter system exists which is cost-efficient, easy to use in clinical laboratories and at the same time allows short turn-around-times.

In our studies we used oncolytic rVACV strains as a model to investigate the potential of a (bacterial) beta-glucuronidase in combination with fluorogenic probes to be used as a general reporter system in biological therapies. The bacterial glucuronidase was chosen as reporter gene, since the pH in blood (pH of about 7.4) is in the pH-optimum range (pH 6.8 - 7.7) of the bacterial enzyme [16] while mammalian beta-glucuronidases are most active at pH 4 to 5 and present almost exclusively in lysosomes. Moreover, the bacterial enzyme displays much higher specific activity compared to the human beta-glucuronidase [30]. On the other hand, bacterial enzymes usually have much higher immunogenicity than their mammalian counterparts and thus might be hindered in their efficacy [31]. Consequently, the group of S. Roffler selected mutants of human beta-glucuronidase with enhanced activity at neutral pH [30] and fused this protein to single chain humanized antibodies for enhanced antibody-directed enzyme prodrug therapy [32]. Therefore, it should also be possible to use this optimized human beta-glucuronidase in the detection system described herein.

Furthermore, the possibility to direct active beta-glucuronidase into the cytoplasm (this study and many others, e.g. [17]), attach it to the cell surface (e.g. [23,33]), or secrete from producing cells (e.g. [32,34]) offers a number of different potential applications.

Cytoplasm associated beta-glucuronidase seems to be a very attractive marker for oncolytic viruses. Not only does presence of glucuronidase in the serum show successful tumor colonization, but also indicates a therapeutic effect: Active enzyme can only get in the serum when (tumor) cells are lysed. Future studies will show if responding and non-responding tumors can be differentiated using the described test system.

The detection of cell surface associated beta-glucuronidase could be helpful in studies relying on transfection of cells or following bacteria or parasite infections in which blood-borne pathogens express a membrane- or cell-wall-anchored glucuronidase. Also, the previously mentioned (non-membrane passing) prodrug therapies would benefit from detection of beta-glucuronidase in the blood: The enzyme would only be observed upon successful prodrug treatment as only then, the active enzyme is released from the tumor.

Secreted beta-glucuronidase could be used as a simple marker for cell survival. Genetically altered cells should secrete active enzyme as long as they are viable. Again, a small number of cells would be sufficient for detection and the amount of beta-glucuronidase in the blood should be correlating with the amount of cells producing the enzyme. Therefore, it will be possible to monitor e.g. stem cell therapies, tissue regeneration etc.

In each case one will have to decide whether the achieved enzyme concentration is sufficient to be detected in the respective assays. Here, we calculated that theoretically only 2.4 × 10^4 ^vaccinia virus infected cancer cells might be enough to be detected in humans. With a diameter of 50 μm/cell this would correspond to a volume of only 1.6 mm^3^. Of course, it can be argued that in patients this number might be higher, as e.g. not all virus infected cells will release all of their enzymes into the blood stream upon lysis. But even if the number of infected cells is 10 or even 100-fold higher, the tumor diameter would only be 3.1 and 6.7 mm, respectively. Nevertheless, only clinical trials such as those in which GLV-1h68 is currently evaluated for safety in human cancer patients [11], will determine whether the proposed detection system can be translated to a clinical setting.

Presumably, similar evaluations will be necessary when applying the presented beta-glucuronidase test in other applications such as in infectious disease studies, stem cell research/therapy etc. both in basic as well as in translational research.

## Conclusions

Taken together, we demonstrated that glucuronidase (in our case encoded by rVACV) in combination with fluorogenic probes has the potential to be used as a general reporter system for heterologous gene expression in biological therapies. Our results provide evidence that the described system cannot only be used for imaging of tumors in the body, but also to confirm viral colonization of these tumors or even the diagnosis of tumors in screening studies.

## List of abbreviations

FBS: fetal bovine serum; FDG: *Fluorescein *di-β-D-galactopyranoside; FDGlcU -fluorescein di-β-D-glucuronide; GFP: Green fluorescent protein; GusA: bacterial glucuronidase; hpi: hours post-infection; LacZ: bacterial beta-galactosidase; MOI: multiplicity of infection; 4-MUG: 4-Methylumbelliferyl-b-D-glucuronide; PFU: plaque forming units; rVACV: recombinant vaccinia virus strain; X-Gal: 5-bromo-4-chloro-3-indolyl-β-D-galactoside; X-GlcU 5-bromo-4-chloro-3-indolyl-β-D-glucuronide.

## Competing interests

The research was supported by the Research and Development Division of Genelux Corp., San Diego, USA, and a Service Grant to the University of Würzburg, Germany also funded by Genelux Corp., San Diego, USA as well as a grant of the "Bundesministerium für Bildung und Forschung" in the MoBiTech initiative (grant number 13N4051). JS, BH, IG and AAS are employees and shareholders of Genelux Corporation and Genelux GmbH respectively. The funders had no role in study design, data collection and analysis, decision to publish, or preparation of the manuscript. No competing interests exist for MH and JBS.

## Authors' contributions

MH and JS conceived the study, designed, performed and analyzed all experiments and wrote the manuscript. JBS and BH participated in live animal studies. IG participated in manuscript writing. AAS participated in conceiving the study and writing the manuscript. All authors read and approved the final version of the manuscript.

## Supplementary Material

Additional file 1 **Figure S1. Viral strains used in this manuscript**. A) Schematic virus constructs. The viral F14.5L, thymidine kinase and hemagluttinin encoding genes of the wild type Lister strain were replaced by the indicated marker genes. B) Verification of marker gene expression by Western blot analysis at 6, 12, 24 and 48 hours post A549 cell infection respectively (multiplicity of infection 0.5). Beta-actin served as loading control. C) X-Gal and X-Gluc staining of single viral plaques. GLV-1h68 encodes both beta-galactosidase and beta-glucuronidase, while control strains lack one or the other gene. Click here for file

Additional file 2 **Figure S2. Time dependent conversion of FDGlcU in the same mouse injected with GLV-1h68**. A A549 tumor-bearing mouse was injected with GLV-1h68 10 days before FDGlcU injection was performed. Intraperitoneal (i.p.) injection (lower row pictures) occurred 24 hours before intravenous (i.v.) injection (upper row pictures). This allowed the fluorescence signal to decline completely before getting the kinetics in the very same mouse. Click here for file

Additional file 3 **Figure S3. Individual mouse data from Figure 4B**. Tumor bearing (red) and non-tumor bearing (grey) control male (upper panels) and female (lower panels) mice were injected with 5 × 10^6 ^pfu GLV-1h68. Analysis of sera revealed conversion of the fluorigenic compounds FDGlcU (left panels) and 4-MUG (right) in all mice. Click here for file

Additional file 4 **Figure S4**. Positive correlation between the fluorescence signal intensities and increasing glucuronidase concentration, fluorogenic substrate concentration (left panels, 4-MUG in A, FDGlcU in B) and incubation time (right panels). Click here for file

Additional file 5 **Figure S5. Glucuronidase assay results independent from presence of human serum**. Increasing amounts of *E. coli *glucuronidase were co-incubated with 4-MUG (upper panel) or FDGlcU (lower panel) in the presence or absence of human serum. Serum samples from 3 different healthy individuals were tested in parallel. **Cover art**. The cover shows the generation of fluorescent products from fluorigenic probes upon cleavage by beta-glucuronidase. From bottom to top decreasing concentrations of beta-glucuronidase were co-incubated with 4-Methylumbelliferyl-b-D-glucuronide, Fluorescein-di-beta-D-glucuronide or without fluorigenic probe in a 384-well plate. The blue compound 4-Methylumbelliferyl and the green Fluorescein were excited using UV-light and photographed without the use of additional emission filters. The very sensitive assay is able to detect picogram amounts of beta-glucuronidase - sufficient for detection of a single beta-glucuronidase expressing cell - as described by Hess et al. in this issue. Click here for file
