# Supplementary figures and images for: Bacterial glucuronidase as general marker for oncolytic virotherapy or other biological therapies
Source: J Transl Med. 2011 Oct 11;9:172. doi: 10.1186/1479-5876-9-172 (PMC3207905; doi:10.1186/1479-5876-9-172)

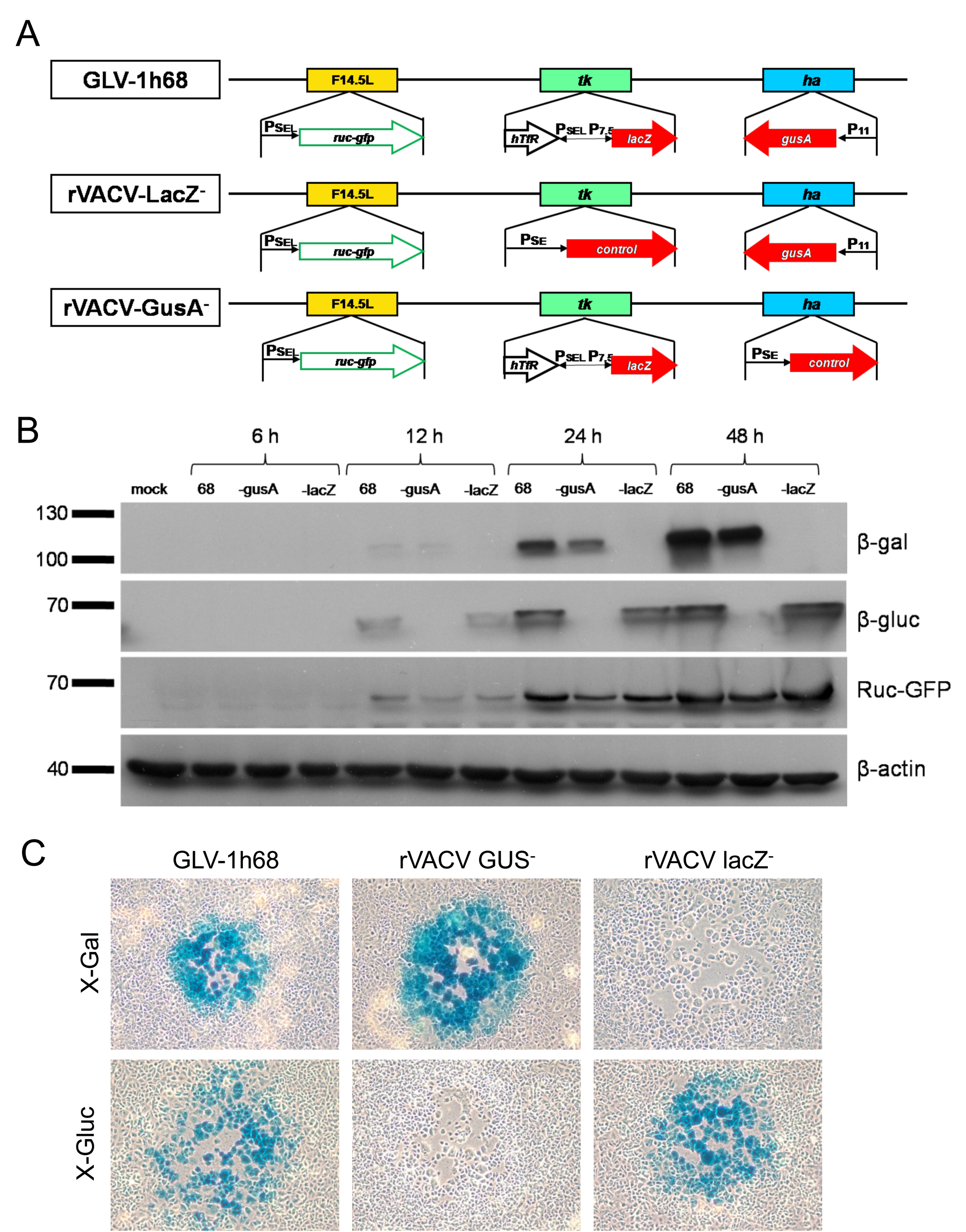

Supplement: Additional file 1 — Figure S1. Viral strains used in this manuscript. A) Schematic virus constructs. The viral F14.5L, thymidine kinase and hemagluttinin encoding genes of the wild type Lister strain were replaced by the indicated marker genes. B) Verification of marker gene expression by Western blot analysis at 6, 12, 24 and 48 hours post A549 cell infection respectively (multiplicity of infection 0.5). Beta-actin served as loading control. C) X-Gal and X-Gluc staining of single viral plaques. GLV-1h68 encodes both beta-galactosidase and beta-glucuronidase, while control strains lack one or the other gene. [file 1479-5876-9-172-S1.TIFF]

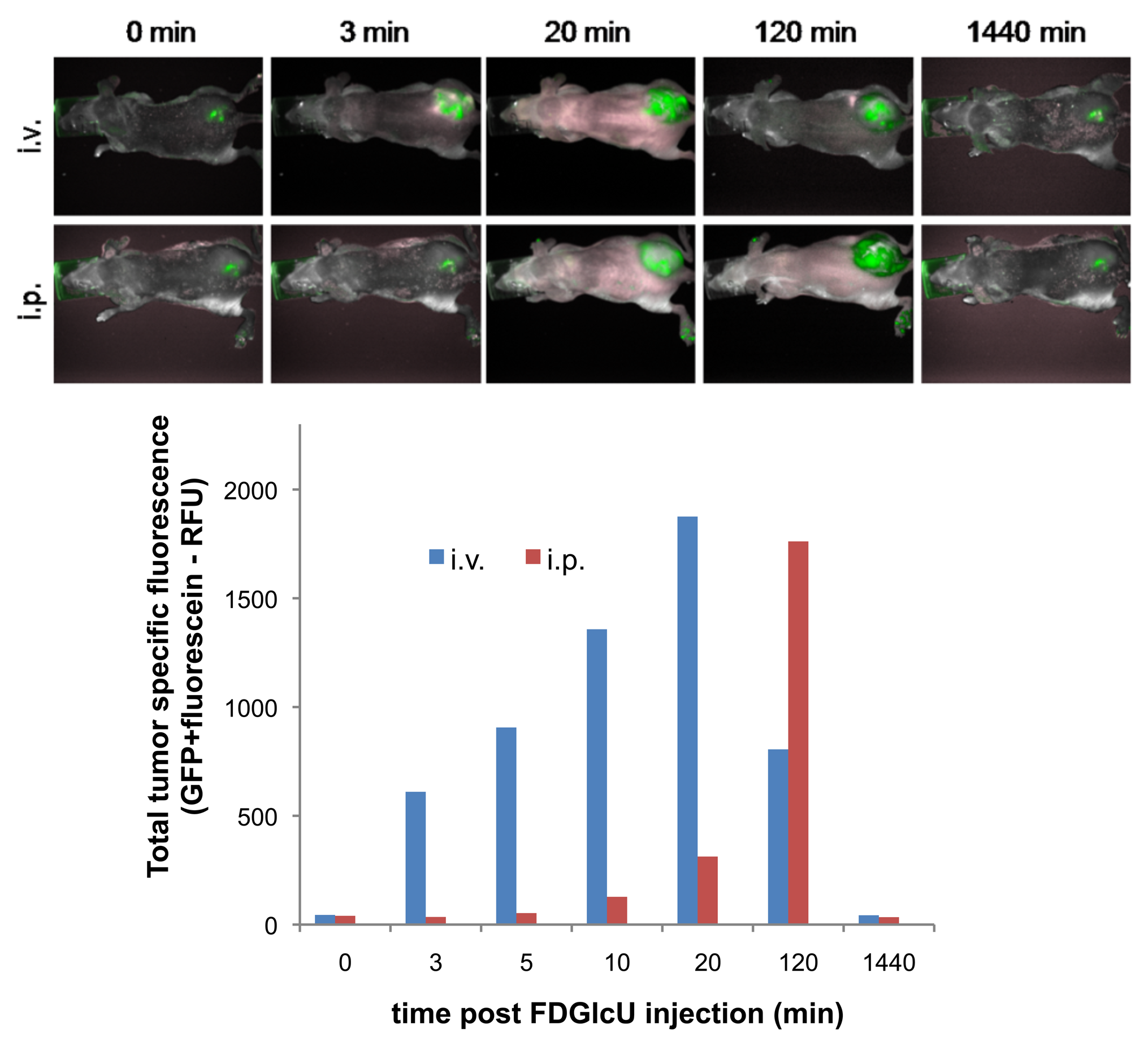

Supplement: Additional file 2 — Figure S2. Time dependent conversion of FDGlcU in the same mouse injected with GLV-1h68. A A549 tumor-bearing mouse was injected with GLV-1h68 10 days before FDGlcU injection was performed. Intraperitoneal (i.p.) injection (lower row pictures) occurred 24 hours before intravenous (i.v.) injection (upper row pictures). This allowed the fluorescence signal to decline completely before getting the kinetics in the very same mouse. [file 1479-5876-9-172-S2.TIFF]

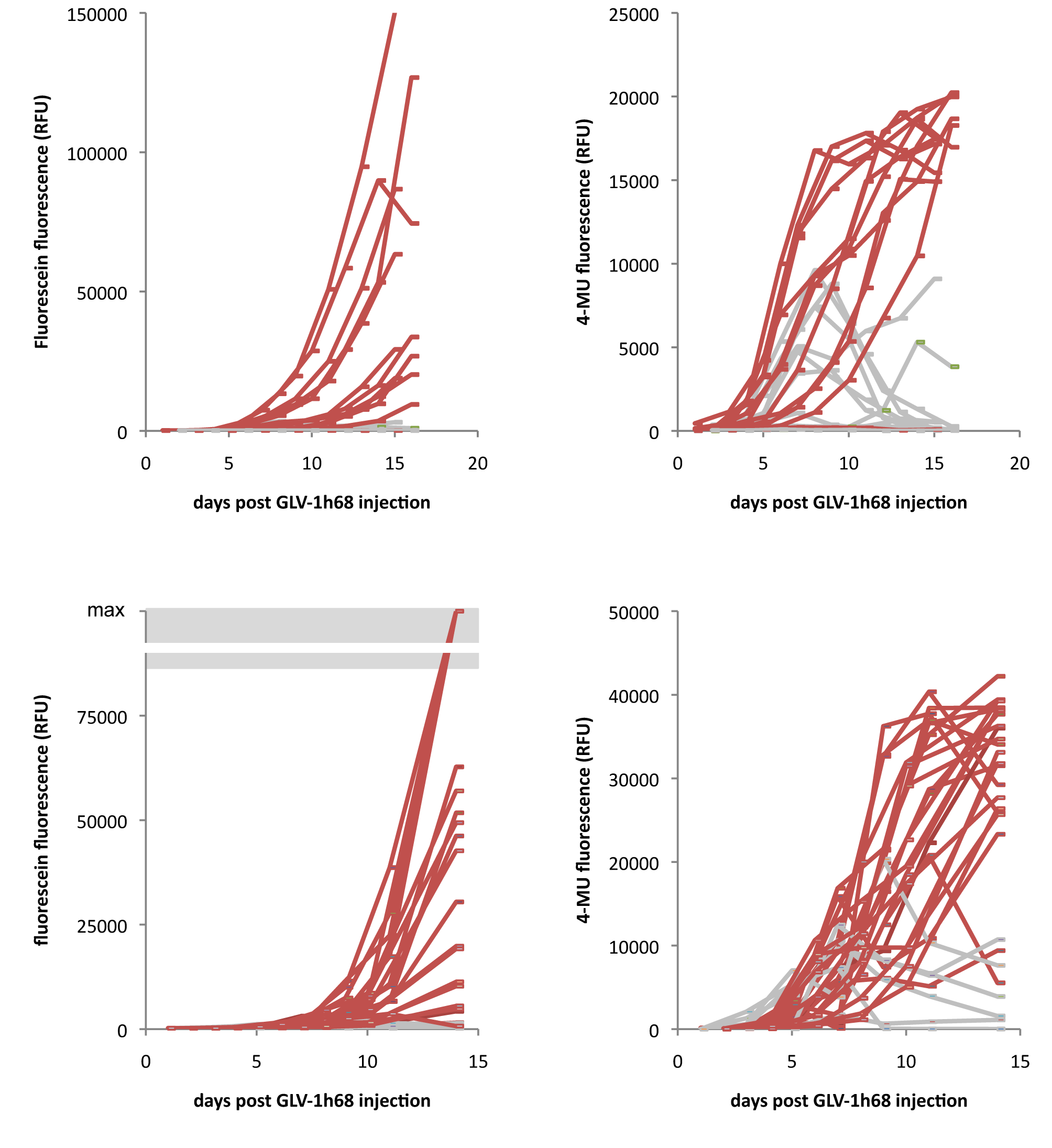

Supplement: Additional file 3 — Figure S3. Individual mouse data from Figure 4B. Tumor bearing (red) and non-tumor bearing (grey) control male (upper panels) and female (lower panels) mice were injected with 5 × 10^6^ pfu GLV-1h68. Analysis of sera revealed conversion of the fluorigenic compounds FDGlcU (left panels) and 4-MUG (right) in all mice. [file 1479-5876-9-172-S3.TIFF]

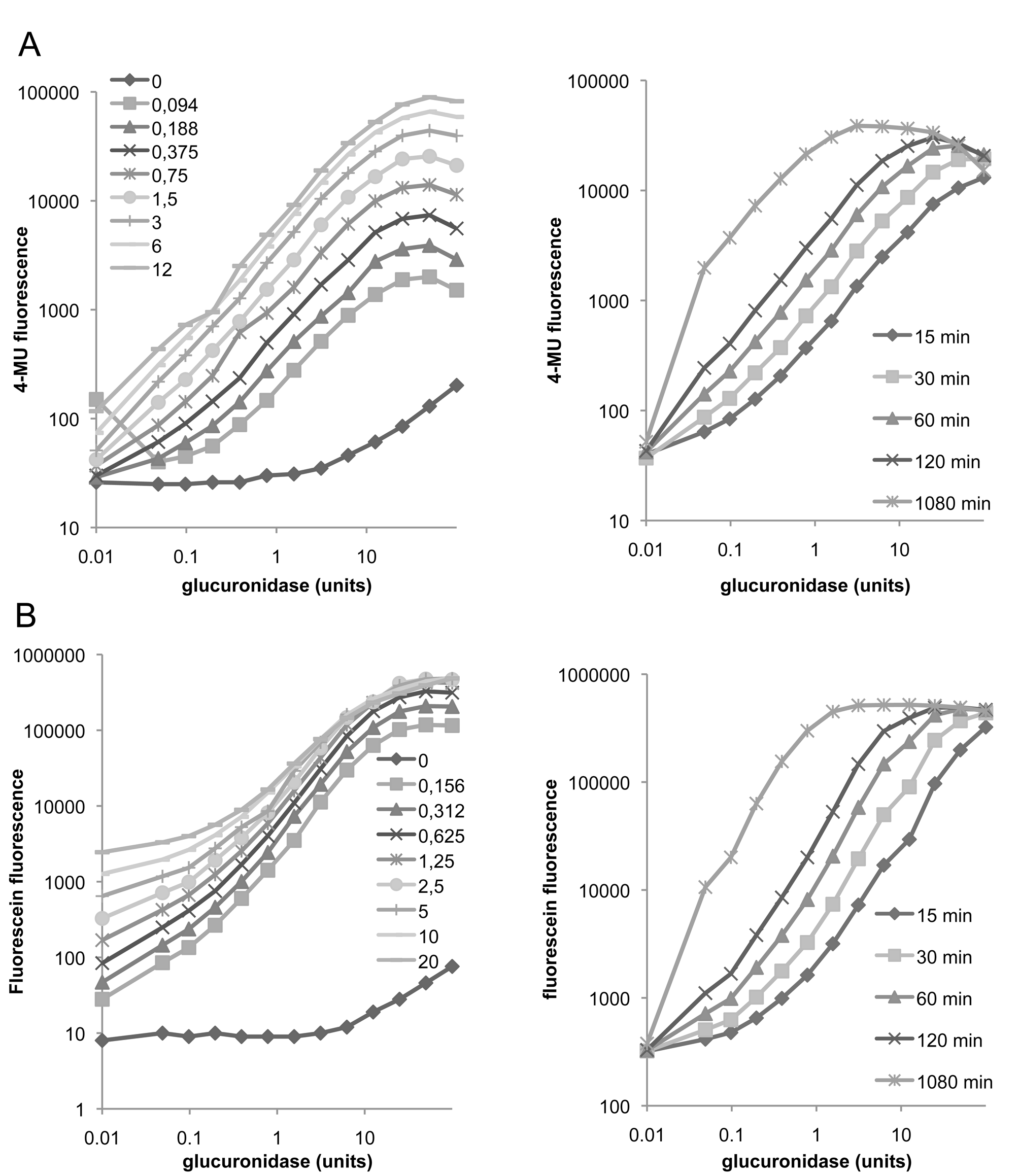

Supplement: Additional file 4 — Figure S4. Positive correlation between the fluorescence signal intensities and increasing glucuronidase concentration, fluorogenic substrate concentration (left panels, 4-MUG in A, FDGlcU in B) and incubation time (right panels). [file 1479-5876-9-172-S4.TIFF]

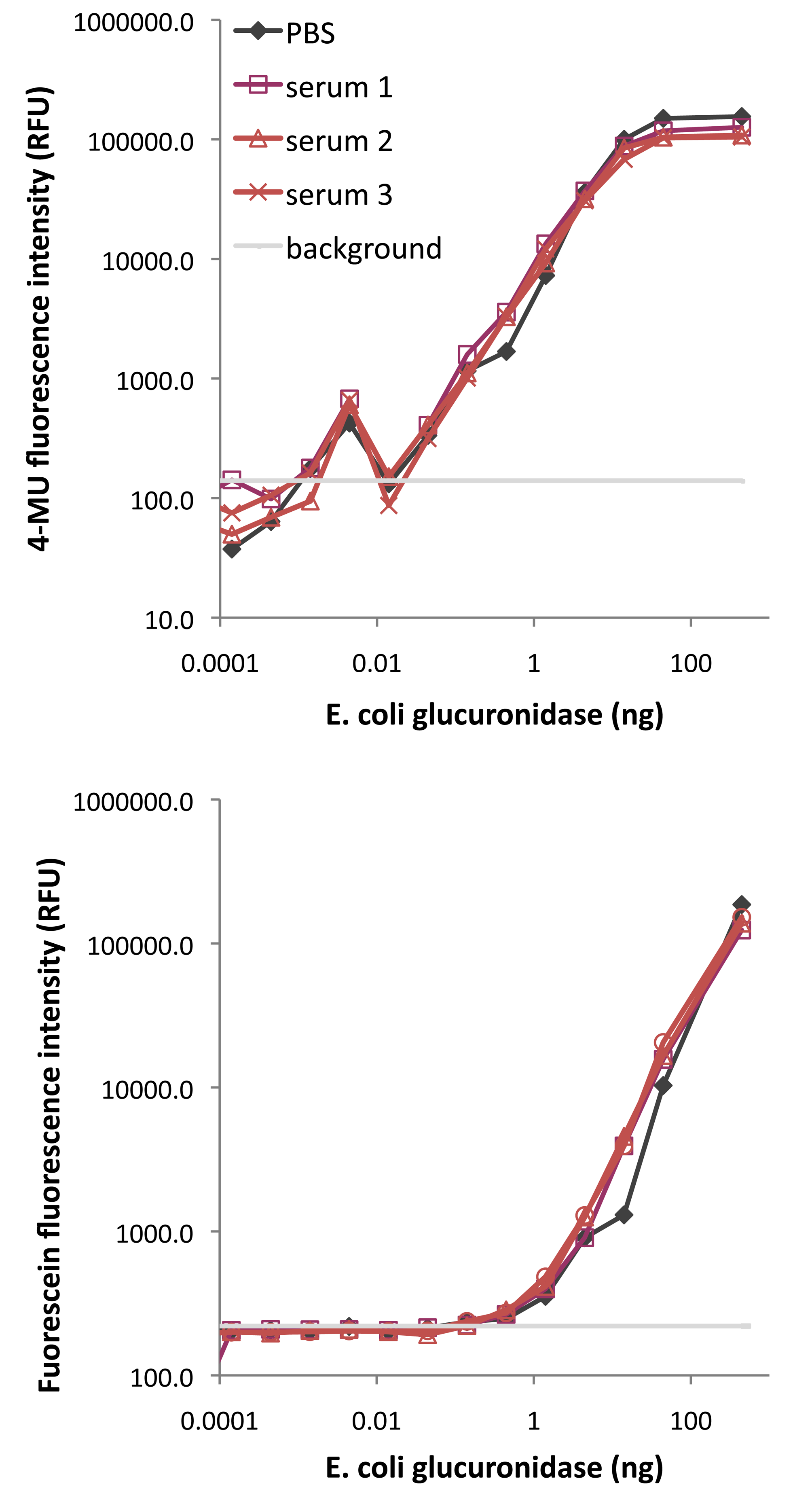

Supplement: Additional file 5 — Figure S5. Glucuronidase assay results independent from presence of human serum. Increasing amounts of E. coli glucuronidase were co-incubated with 4-MUG (upper panel) or FDGlcU (lower panel) in the presence or absence of human serum. Serum samples from 3 different healthy individuals were tested in parallel. Cover art. The cover shows the generation of fluorescent products from fluorigenic probes upon cleavage by beta-glucuronidase. From bottom to top decreasing concentrations of beta-glucuronidase were co-incubated with 4-Methylumbelliferyl-b-D-glucuronide, Fluorescein-di-beta-D-glucuronide or without fluorigenic probe in a 384-well plate. The blue compound 4-Methylumbelliferyl and the green Fluorescein were excited using UV-light and photographed without the use of additional emission filters. The very sensitive assay is able to detect picogram amounts of beta-glucuronidase - sufficient for detection of a single beta-glucuronidase expressing cell - as described by Hess et al. in this issue. [file 1479-5876-9-172-S5.TIFF]
